# Production of New Microbially Conjugated Bile Acids by Human Gut Microbiota

**DOI:** 10.3390/biom12050687

**Published:** 2022-05-11

**Authors:** Carlos J. Garcia, Vit Kosek, David Beltrán, Francisco A. Tomás-Barberán, Jana Hajslova

**Affiliations:** 1 Department of Food Analysis and Nutrition, Faculty of Food and Biochemical Technology, University of Chemistry and Technology, 16628 Prague, Czech Republic; hernandc@vscht.cz (C.J.G.); vit.kosek@vscht.cz (V.K.); 2 Laboratory of Food and Health, CEBAS-CSIC, Food Sci. & Technology Deptartment, Campus Universitario de Espinardo-25, E-30100 Murcia, Spain; dbeltran@cebas.csic.es (D.B.); fatomas@cebas.csic.es (F.A.T.-B.)

**Keywords:** metabolomics, gut microbiota, bile acids, MCBAs

## Abstract

Gut microbes have been recognized to convert human bile acids by deconjugation, dehydroxylation, dehydrogenation, and epimerization of the cholesterol core, but the ability to re-conjugate them with amino acids as an additional conversion has been recently described. These new bile acids are known as microbially conjugated bile acids (MCBAs). The aim of this study was to evaluate the MCBAs diversity produced by the gut microbiota through a metabolomics approach. In this study, fresh fecal samples from healthy donors were evaluated to explore the re-conjugation of chenodeoxycholic and 3-oxo-chenodeoxycholic acids by the human gut microbiota. No significant differences were found between the conversion trend of both BAs incubations. The in vitro results showed a clear trend to first accumulate the epimer isoursochenodeoxycholic acid and the dehydroxylated lithocholic acid derivatives in samples incubated with chenodeoxycholic and 3-oxo-chenodeoxycholic acid. They also showed a strong trend for the production of microbially conjugated dehydroxylated bile acids instead of chenodeoxycholic backbone conjugates. Different molecules and isomers of MCBAs were identified, and the new ones, valolithocholate ester and leucolithocholate ester, were identified and confirmed by MS/MS. These results document the gut microbiota’s capability to produce esters of MCBAs on hydroxyls of the sterol backbone in addition to amides at the C24 acyl site. This study opens a new perspective to study the BAs diversity produced by the human gut microbiota.

## 1. Introduction

Bile acids (BAs) are terpenoids biosynthetized by the human body. BAs are produced from cholesterol in the hepatocytes, stored in the gall bladder, and released into the small intestine [1]. Specifically, BAs are endogenous acidic steroids with amphiphilic properties [2]. The conversion of cholesterol to BAs entails complex pathways including hydroxylations, saturation of double bonds, epimerizations and oxidations [3]. The BAs produced are known as primary BAs, including cholic acid (CA) and chenodeoxycholic acid (CDCA).

BAs carry out important functions in fat absorption thanks to their amphiphilic nature. Their role is key in the lipid metabolism. BAs form mixed micelles in the small intestine that facilitate solubilization, digestion, and the absorption of dietary lipids and fat-soluble vitamins. Micelles solubilize cholesterol in bile preventing cholesterol crystallization which may prevent gallstone formation [4]. BAs are also signaling molecules and metabolic regulators that activate nuclear receptor and G protein-coupled receptor signaling to regulate hepatic lipids, glucose, and maintain metabolic homeostasis [5]. Therefore, these signaling functions could affect the health of the host [6].

The original set of BAs depends of the host biosynthesis. However, the final BAs pool is significantly influenced by the gut microbiota. Primary BAs are converted into the secondary bile acids by the gut microbes and, consequently [7], this conversion should be considered a very important factor in the BAs homeostasis [8]. The gut microbiota is known to perform deconjugations of conjugated primary BAs by bile salt hydrolase (BSH), dehydroxylations by 7α-dehydroxylase, and oxidations by hydroxysteroid dehydrogenase activities [9]. In addition to these previous conversions, recent studies have shown the ability of gut microbiota to re-conjugate BAs with amino acids producing compounds known as microbially conjugated bile acids (MCBAs) [10]. These metabolites were characterized as amide conjugations in the C24 acyl site similarly to those of the original primary BAs with glycine and taurine. The MCBAs have been associated with the bacteria *Enterocloster bolteae* and *Clostridium bolteae* as the main producers of these BAs. The mechanism through which the gut microbes mediate this conjugation is still unknown, but it is thought that it could be similar to the mechanism of amino acid N-acyltransferase (BAAT) [11], an enzyme located in hepatocytes which is in charge of the production of bile salts such as taurocholic acid, glycocholic acid, taurochenodeoxycholic acid and glycochenodeoxycholic acid.

The understanding of the BAs pathophysiology has been the goal of many studies in recent years which have demonstrated the relevance of the BAs and the microbiota in the development of very important diseases [12,13,14]. The relationship between BAs and the gut microbiota has been associated with important diseases [15]. BA diarrhea (BAD) is associated with BAs and microbiota interactions leading to dysbiosis [16]. Similarly, dysbiosis has been associated with Parkinson’s disease [14]. A multi-omics study including 123 subjects affected with inflammatory bowel disease showed an increase in both the BAs and in facultative anaerobes [17,18]. Therefore, the present study of the production of the new microbially conjugated bile acids (MCBAs) is a necessary approach to characterize the microbial BAs diversity and to go one-step further in the knowledge of the BAs pathophysiology. The presence of these new re-conjugated compounds would compromise the initial bile salt hydrolase (BSH) function of releasing free BAs for further conversions, thus severely modifying the BAs pool. Delving into the metabolomic techniques for the identification of these compounds as addressed in this research is a key approach to elucidating the complete family of bile acids corresponding to these new conversions produced by the human gut microbiota and to deepen into the knowledge of its biological activity.

The aim of this study was to apply a sensitive analytical method to identify secondary BAs and new microbially conjugated bile acids ((MCBAs) by ultra-high-performance liquid chromatography coupled to mass spectrometry (UHPLC-MS/MS). Metabolomic platforms have shown to be a very adequate tool for identification and confirmation of primary and secondary BAs [19]. This study uses metabolomics to evaluate the classical conversions and the new re-conjugations of BAs by human gut microbiota. Targeted and untargeted metabolomics have been used satisfactorily in other in vitro studies to describe the human gut microbiota metabolism of plant sesquiterpene lactones [20]. The final goal of this in vitro study was evaluating the conversion of BAs by the human gut microbiota in order to describe the microbially conjugated BAs diversity.

## 2. Materials and Methods

### 2.1. Chemicals

Authentic standards of 3α,7α-Dihydroxy-5β-cholan-24-oic Acid (chenodeoxycholic acid) and 3α-Hydroxy-11-oxo-5β-cholan-24-oic Acid (3-oxo-chenodeoxycholic acid) were purchased from Avanti Polar Lipids (Alabaster, AL, USA). Nutrient Broth (NB) was from Oxoid (Basingstoke, Hampshire, UK). l-Cysteine hydrochloride (Panreac Química, Barcelona, Spain).

### 2.2. Collection of Human Fecal Samples

Two healthy donors of stool samples, one male (age 44) and a female (age 30), were recruited at the Centro de Edafología y Biología Aplicada del Segura (CEBAS-CSIC, Murcia, Spain) and both gave written informed consent. The sample evaluation of two donors is adequate to describe the presence and confirmation by metabolomics of secondary and conjugated BAs, providing an appropriate number of samples to certify detection. Each volunteer provided a fresh stool sample early in the morning to perform the fermentation experiments. Samples were stored at 4 °C and further processed within one hour of donation.

Institutional ethical approval was not necessary as the experiments were did not constitute an intervention study, and only fecal samples were collected, as advised by the Catholic University of Murcia (Murcia, Spain) Ethical Committee. The volunteers gave written informed consent.

### 2.3. Conversion Experiments by Human Fecal Cultures

Preparation of fecal suspensions and subsequent fermentation experiments were performed under anoxic conditions in an anaerobic chamber (Concept 400, Baker Ruskinn Technologies, Ltd., Bridgend, South Wales, UK) with an atmosphere consisting of N_2_/H_2_/CO_2_ (85:5:10) at 37 °C. Aliquots of stool samples (10 g) were diluted 1/10 *w*/*v* in Nutrients Broth supplemented with 0.05% l-cysteine hydrochloride and homogenized by a stomacher in filter bags. Four types of samples were used: (i) BAs + fecal samples incubated; (ii) fecal samples incubated (control 1); (iii) BAs + fecal samples non-incubated; and (iv) fecal samples non-incubated (control 2). Aliquots of fecal suspensions (50 µL) were inoculated into 5 mL of fermentation medium anaerobe Wilkins Chaldean containing either 50 µm of chenodeoxycholic acid (CDCA) or 50 µm 3-oxo-chenodeoxycholic acid (3-oxo-CDCA). The chenodeoxycholic acid (CDCA) was the primary BA selected due his medium hydrophobicity and therefore his medium associate toxicity, being the study able to evaluate how the microbiota conversions modulate the initial hydrophobicity. The oxo-BA, 3-oxo-chenodeoxycholic acid (3-oxo-CDCA) was used in order to evaluate the conversion trend to produce dehydroxylateds BAs or oxo-BAs. Three replicate cultures were prepared in parallel from each fecal suspension. Two types of fecal control samples were prepared, one including incubation and one without the incubation procedure. Fecal sample controls and either CDCA and 3-oxo-CDCA (5 mL) were incubated with fecal microbiota and samples were collected after five days of incubation at 37 °C. The duration of the fecal incubation was set in order to ensure conversion procedures by the gut microbiota. Usually after 24–48 the bacteria are already in a stationary phase and it is in this phase when the secondary metabolism, which acts in this conversion, commonly occurs [21]. After the incubation, samples were extracted with 5 mL of ethyl acetate LC-MS (Scharlau, Barcelona, Spain) using a refrigerated thermoblock shaker (VWR lnternational, LLC, Radnor, PA, USA) at 20 °C for 10 min at 1500× *g* rpm. Samples were centrifuged at 3500× *g* for 10 min at 4 °C. The organic phase was evaporated under reduced pressure in a speed vacuum concentrator (Savant SPD121P, Thermo Scientific, Waltham, MA, USA). All of these procedures were performed at CEBAS-CSIC (Murcia, Spain). Extracts were then transferred to the Department of Food Analysis and Nutrition (University of Chemistry and Technology, Prague) for instrument analysis. Samples were re-dissolved in 500 µL of methanol, and filtered through a 0.22 µm PVDF filter (Merck Millipore, Cork, Ireland), and they were diluted 1:2 in methanol before the UPLC-IM-QTOF-MS analysis.

### 2.4. UPLC-IM-QTOF-MS Analysis

A metabolomics analysis was performed on a U-HPLC (Infinity 1290; Agilent, Santa Clara, CA, USA) coupled to a high-resolution mass spectrometer with a hyphenated quadrupole time-of-flight mass analyzer (6560 Ion Mobility Q-TOF LC/MS; Agilent) with an Agilent Jet Stream (AJS) electrospray (ESI) source. The mass analyzer was operated in negative mode under the following conditions: gas temperature 180 °C, drying gas 12 L/min, nebulizer pressure 45 psig, sheath gas temperature 350 °C, sheath gas flow 11 L/min, capillary voltage 3500 V, nozzle voltage 250 V, fragmentor voltage 350 V, and octapole radiofrequency voltage 250 V. Data were acquired over the *m*/*z* range of 50–1700 at the rate of 2 spectra/s. The *m*/*z* range was autocorrected on reference masses 119.0363 and 980.0164.

The chromatographic analysis was mostly replicated from Reiter et al. 2021, equipped with 100 × 2.1 mm, 100 Å, 1.7 μm, Kinetex C18 column (Phenomenex, Aschaffenburg, Germany) and performed at a constant flow rate of 0.4 mL/min. Mobile phases consisted of water (A) and acetonitrile/water (95/5, *v*/*v*) (B), both containing 5 mM ammonium acetate and 0.1% formic acid. The gradient started with 25% B for 2 min, increased in 1.5 min to 27% B, in 2 min to 35% B, held for 4.5 min, increased in 1 min to 43% B, held for 2 min, increased in 2 min to 58% B, held for 3 min, increased in 0.5 to 65% B, in 0.5 min to 80% B, in 1 min to 100% B, held 1 min at 100% B, and decreased to the initial conditions followed by 2 min of re-equilibration. The injection volume for all samples was 3 µL, the column oven temperature was set at 40 °C.

### 2.5. Metabolomics Data Treatment

The data pre-processing was performed on all samples using Profinder software (Agilent Technologies, Santa Clara, CA, USA) in negative polarity mode. The pre-processing operations were set in seven configurations under the Molecular Feature Extraction (MFE) batch for small molecules. The operations included summary extraction parameters (*m*/*z* and RT restriction and height spectra limitation), compound binning and alignment (mass and RT tolerance), feature filter (absolute height), extract ion tolerance (EIC), EIC peak integration (Agile2 algorithm, Gaussian smoothing function, spectrum extraction) and spectrum extraction. The pre-processing operation gave a data matrix based on 10806 entities from a full data set. The data matrix was imported to Mass Professional Profiler (MPP, Agilent technologies) for processing including log transformation and Pareto scaling [22].

A specific database was created according to a generic file format, for all possible MCBA combinations. The database was built using PDCL software (Agilent Technologies) to allow its use in the Agilent software package environment.

ANOVA (corrected *p*-value cut-off: 0.05; *p*-value computation: Asymptotic; Multiple Testing Correction: Benjamini-Hochberg) statistics analysis and Fold change tools were used for filtering entity candidates which were not present in the control and were detected in the samples after the incubation with BAs.

## 3. Results

Four different pathways of microbial conversion of bile acids are conventionally known: deconjugation, dehydroxylation, dehydrogenation, and epimerization. The present study will refer to these conversions as classical microbial conversions. Deconjugation of BAs is considered the opening reaction for the rest of the modifications and its relevance is explained due the fact that deconjugated primary BAs can act as signaling molecules which modify the total bile acid pool, and therefore the gut microbes may incorporate the deconjugation mechanism to modify bile production [23]. The deconjugation is performed by the bile salt hydrolases (BSHs). Enzymes capable of carrying out deconjugation have been found in most bacterial phyla, and all BSH reactions are based on amide bond hydrolysis in order to free taurine or glycine [24]. The dehydroxylation of the unconjugated BA is a multi-stage process that includes substrate oxidation, probably for molecule stability prior to dehydroxylation, followed by the reduction at each previously oxidized position along the sterol backbone [25]. The last two mechanisms of BA conversions act together, since the formation of oxo-BA is a key step before epimerization. Two steps are required for epimerization conversion: oxidation of the hydroxyl group by a position-specific hydroxysteroid dehydrogenase, such as a 7α-HSDH, followed by the reduction of another position-specific hydroxysteroid dehydrogenase, 7β-HSDH. These reactions do not necessarily have to be carried out by the same microorganism but can be a co-culture of several, giving rise to a long chemical diversity of secondary BAs [26]. In addition to the classical conversion, this study evaluated the new re-conjugations conversions leading to the compounds known as “microbially conjugated bile acids” (MCBAs). These recently discovered BAs conjugated at the C24 acyl site similar to the original host conjugation mechanism but instead to the classical ones conjugated with taurine or glycine, were conjugated with the amino acids phenylalanine, leucine, and tyrosine on a cholic acid backbone [27]. This study will refer to this new conversion as microbially conjugated bile acids.

The classical microbial conversion of BAs was evaluated in advance, as both the classical metabolism and the novel amino acid conjugations could compete for the sterol backbone of the incubated BAs.

### 3.1. Classical Microbial Conversion of Bile Acids

The primary BAs CDCA and the secondary oxo-bile acid 3-oxo-CDCA were fermented in vitro to individually evaluate their conversion by the volunteers’ gut microbiota.

**Figure 1:**
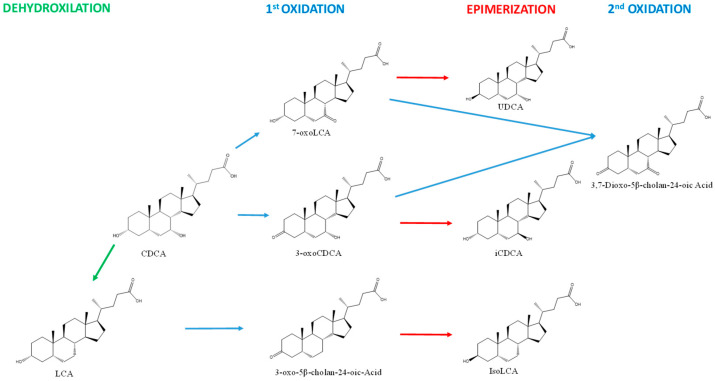
Summary of the expected conversion of secondary BAs by human gut microbiota.

The classical microbial conversions expected according to BAs used for the incubation included the dehydroxylation, dehydrogenation, and epimerization of CDCA and 3-oxo-CDCA [8,9,28] (Figure 1).

**Figure 2:**
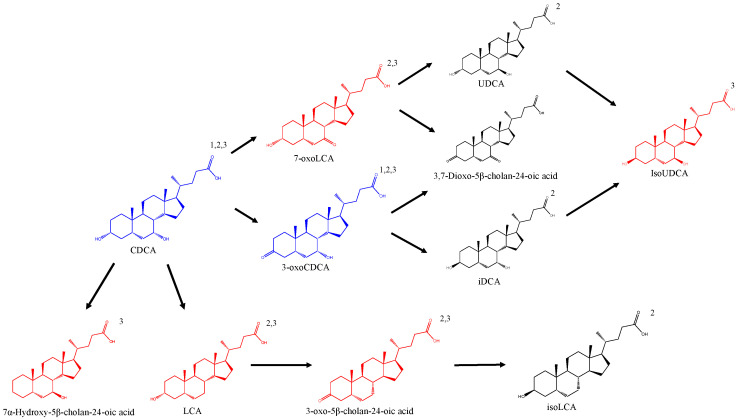
BAs trend identified after samples incubation. Black color: non-detected; Red color: detected after incubation; Blue color: incubated and detected. CDCA: Chenodeoxycholic acid; LCA: Lithocholic acid; UDCA: Ursodeoxycholic acid; iDCA Isodeoxycholic acid. 1: Identified with authentic standard; 2: Identified according retention time based on Reiter et al., 2021; 3: Identified by MS/MS fragments.

No significant differences were found for both BAs, with epimerization and dehydroxylation being the most significant metabolic changes. A total of six secondary BAs were identified and confirmed by MS/MS analysis. The epimer identified as isoursochenodeoxycholic acid (iUDCA), in addition to lithocholic acid and 3-oxo-5β-cholan-24-oic acid, was the most prominent BA produced after incubation (Figure 2) (Table 1).

The iUDCA identified expressed 2.7× 10^7^ of absolute abundance, while its precursor CDCA, incubated with a concentration of 50 µM, gave a signal response of 1.39× 10^9^. Therefore, attending the signal response differences, the results showed the relatively relevant production of the epimer. The common expected epimers ursodeoxycholic acid (UDCA), produced via 7α/β-HSDH, and isodeoxycholic acid (iDCA), produced via 3α/β-HSDH, were not observed following the analytical data provided by the referenced method [18]. Lithocholic acid (LCA), 7α-Hydroxy-5β-cholan-24-oic acid, and 3-oxo-5β-cholan-24-oic acid presented absolute abundances of 9.5 × 10^7^, 1.4 × 10^7^ and 1.73 × 10^7^, respectively. No epimers were detected for the monohydroxylated BAs lithocholic acid and 7α-Hydroxy-5β-cholan-24-oic acid. The oxo-BAs 3-oxoCDCA, 7-oxoLCA, and 3-oxo-5β-cholan-24-oic acid were identified. The 3,7-Dioxo-5β-cholan-24-oic acid was not detected. The results showed an increase of 3-oxoCDCA and 7-oxoLCA, in case of sample incubation with CDCA, but an increment of 7-oxoLCA in the case of incubation with 3-oxoCDCA was also detected. These results can be explained due the fact that an increment of CDCA was detected in samples incubated with 3-oxoCDCA by the reversible action of 3α/β-HSDH.

### 3.2. Production of Microbially Conjugated Bile Acids (MCBAs)

Once the classical microbial conversions were evaluated, the in vitro fermentation of the secondary BA CDCA and the secondary oxo-bile acid 3-oxo-CDCA were used to investigate the conversion trends of MCBAs by the gut microbiota.

**Figure 3:**
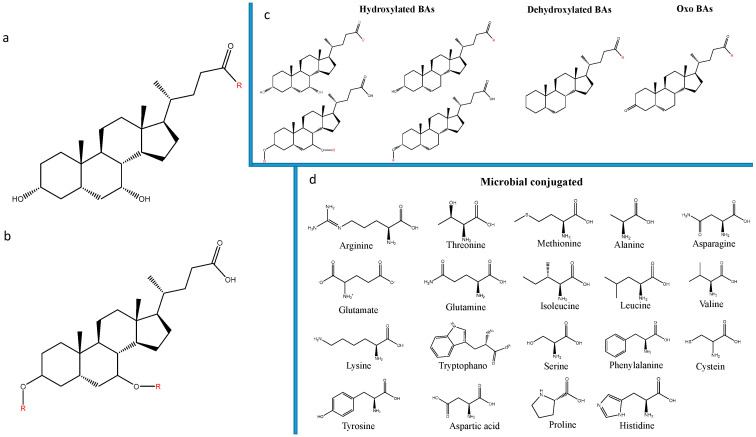
Conversion possibilities of microbially conjugated BAs by the human gut microbiota. (**a**) Position possibilities for the amino acid along the cholic acid backbone; (**b**) Position possibilities for the amino acid along the cholic acid backbone in case of esterification reaction; (**c**) Position possibilities for the amino acid in case of hydroxylated, dehydroxylated and oxidized BAs; (**d**) Amino acid used to cover the conjugation possibilities.

The MCBAs were investigated by using an in-house database built and based in all the possible calculated conversions (Figure 3).

The database included, in addition to BAs re-conjugated in the 24-acyl site, the mono and di esters of MCBAs on the 3 and 7 hydroxyls. According to this, a total of 226 molecules were calculated and searched.

Those ions that were detected in incubated samples, undetected in control samples and matched in the MCBAs database were filtered and selected as candidates for further evaluation. No significant differences were found for both BA incubations in agreement with the classical trend of conversion. The results showed a higher accumulation of conjugated dehydroxylated BAs instead of the conjugates derived from the CDCA backbone. Eighteen MCBAs were identified after incubation with secondary BAs and only one was tentatively identified as a conjugate derived from chenodeoxycholic acid (Table 2).

**Figure 4:**
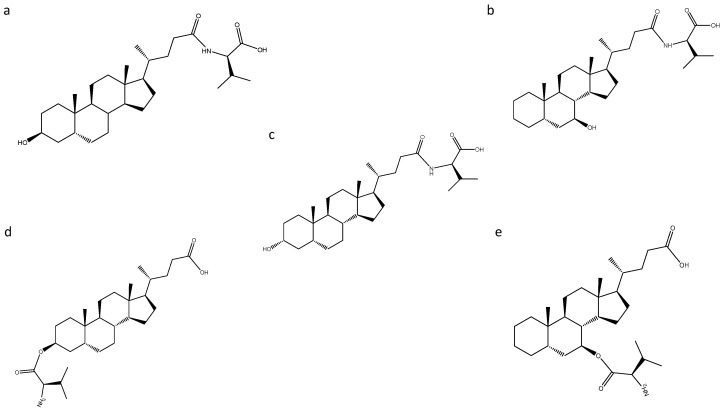
MCBAs of valine identified. (**a**) valoisolithocholic acid; (**b**) valoiso7β-Hydroxy-5β-cholan-24-oic acid; (**c**) valolithocholic acid; (**d**) valoisolithocholate ester; (**e**) valoiso7β-Hydroxy-5β-cholate ester.

None of the MCBAs identified were detected in the control samples. The MCBAs are not detected in the control samples probably because the BAs can be reabsorbed in the terminal ileum and transported to the liver. This enterohepatic recirculation is very effective, and approximately 95% of secreted bile acids including some of the ones modified by the gut microbiota are recirculated [29]. Of the total of eighteen MCBA identified, ten of them were tentatively identified. It was not possible to increase this degree of identification due to the intensity of the ion in its fragmentation and the impossibility of increasing the injection volume due to the small amount of the biological sample. On the other hand, eight MCBAs were confirmed through MS/MS fragments by the detection of the loss of the amino acid on the bile acid steroid backbone, similar to the first identification of these novel compounds [27]. As an additional comparison, the MS/MS spectra calculated in negative mode by competitive fragmentation modelling for metabolite identification (CFM-ID software platform) of fatty acids conjugated with valine as N-palmitoyl valine, N-linoleoyl valine and N-stearoyl valine present major fragment of loss of valine (*m*/*z* 116.0717), which in agreement with our results. Five and three conjugated molecules of valine and leucine were identified and confirmed by MS/MS, respectively. The identification of the five valine-conjugated molecules showing an equal *m*/*z* prompted us to study the occurrence of conjugates derived from hydroxyl esterifications as possible new molecules since the valine conjugate, identified as valolithocholic, can only yield three isomers of these molecules according to the previous classical conversion results. With regard to this, the valine conjugates were identified as valolithocholic acid and valolithocholate ester (Figure 4).

**Figure 5:**
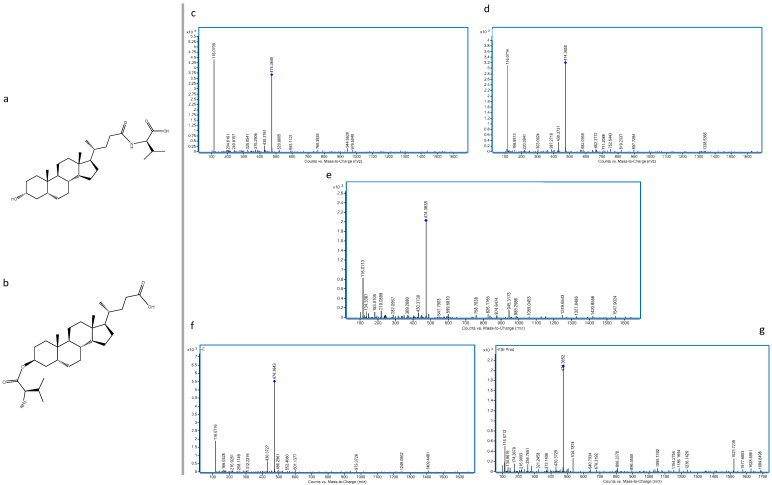
MS/MS spectra of valine conjugates. (**a**) microbially conjugated BA in the 24 acyl site; (**b**) microbially conjugated BA by esterification of C3; (**c**) MS/MS fragments of valine conjugate confirmed at 17.15 min; (**d**) MS/MS fragments of valine conjugate confirmed at 16.80 min; (**e**) MS/MS fragments of valine conjugate confirmed at 16.16 min; (**f**) MS/MS fragments of valine conjugate confirmed at 15.48 min; (**g**) MS/MS fragments of valine conjugate confirmed at 14.83 min.

The MS/MS analysis was not able to discriminate between both molecules due the fragmentation spectra similarity (Figure 5).

In the case of leucine conjugates, leucolithocholic, isoleucolithocholic and leucocholate ester were identified as possible molecules, and similarly the MS/MS analysis was not able to discriminate between them.

The impact of the conjugations is clear since the addition of the amino acids changes the BA chemical properties. In case of the valine conjugated metabolites, being valine an hydrophobic amino acid, the conjugates showed an decrease in the retention time with respect to LCA, which suggested that these valine conjugates may be related to the epimer of LCA, isoLCA (Figure 1). This result agrees with the classical conversion pathways where isoLCA was not detected and in case of isoLCA the retention time must be less with respect to the detected lithocholic acid according to the referenced method [18]. The results showed a decrease in the retention time of all valine conjugates when they were compared with that of isoLCA. These findings suggest that isoLCA was not detected by the classical conversion because it underwent conjugation by the gut microbiota and therefore was not detected as an unconjugated molecule. In addition, it has been described that the amidation at the 24-acyl site, which occurs in the hepatocytes during glycine conjugation, increases hydrophilicity of the molecule, and this may also explain the variation in the retention time [30]. With regard to this, we propose that the conjugates valo-isolithocholic acid (Figure 4a), valo-7β-Hydroxy-5β-cholan-24-oic acid (Figure 4b), valo-lithocholic acid (Figure 4c), valo-isolithocholate ester (Figure 4d), and valo-7β-Hydroxy-5β-cholate ester (Figure 4e) were the metabolites identified. In the case of leucine, which is a hydrophobic amino acid contributing to the increase of hydrophobicity, the results showed an increase in the retention time of two leucine conjugated BAs with respect to non-conjugated lithocholic acid (Table 1 and Table 2). Therefore, in the case of leucine only one of them was consider as an epimeric conjugate.

The esterification of BAs in the gut, performed by *Bacteroides*, has been known for several years [7]. Polyesters of DCA and long-chain fatty acid esters of LCA have been described [31,32]. It has been calculated that the esterified BAs may represent more than about 25% of the total fecal BAs. This is relevant since esterification increases the hydrophobicity of these molecules, but there is no previous description of how esters of amino acids are affected. Despite its importance, not much is known about the role of gut microbiota in these reactions [33].

Other MCBAs were tentatively identified after the incubation, and as described above, some of them presented different molecular and isomeric possibilities. Leucochenodeoxycholic (isoleucolithocolic; leucolithocholate ester), prololithocholic (prololithocholate; valo-oxolithocholic) alanolithocholic (alanolithocholate ester, serocholic acid, serocholate ester) and arginolithocholic (arginolithocholate ester), lysocholic acid (lysocholate ester) and threonocholic acid (threonocholate ester) were detected.

The precise mechanism through which gut microbes perform the different conjugations proposed in this study has yet to be described, although to describe the conjugation in the 24-acyl site, a similar mechanism to that of hBAAT within the liver involving a Cys-Asp-His triad has been suggested, with cysteine functioning as the catalytic residue for nucleophilic attack [10].

## 4. Discussion

In summary, the classical conversion is based on the production of the epimeric isoursodeoxycholic (IsoUDCA) and lithocholic acids even when the reaction starts with the oxidized BA. Leaving aside the dehydroxylation reactions, the trend is the accumulation of the epimer instead of the oxidized form. On the other hand, in the case of dehydroxylated derivatives, the accumulation of the oxidized BAs is relevant.

These results were significant since the epimerization causes an alteration of the hydrophilicity of BAs [34]. This change in solubility affects the efficiency of lipid solubilization or the affinity for hydroxysteroid dehydrogenases. For this reason, all the hydroxy groups of BAs synthesized in the hepatocytes have an α orientation, introducing an amphiphilic behavior to the BAs and allowing the efficient solubilization of lipids [35]. Furthermore, the epimerization trend by the gut microbiota and the consequent accumulation of hydroxy groups with a β-orientation in the final BAs pool confers a protective effect on the liver against the more toxic, hydrophobic bile acids [36]. BA hydrophobicity is an important determinant of the toxicity and protection of BAs. BA hydrophobicity depends on the number, position, and orientation of the hydroxyl groups, as well as the amidation at the C-24 position [29]. Thus, this result suggests that epimerization reduces the toxicity level of the final pool of BAs, reducing the molecules with α orientation hydroxyl groups and the production of dehydroxylated BAs due to the limitation of the affinity of hydroxysteroid dehydrogenases.

Accordingly, the correct performance of lipid absorption functions by BAs and their toxicity are affected by the microbial community of the host based on its ability to perform specific BA transformations. The classical conversion trend of secondary BAs may affect the final conversion into MCBAs.

Regarding the new microbially conjugated bile acids, the production of MCBAs derived from CDCA and 3-oxo-CDCA was not as relevant as the accumulation of the epimer iUCDA, which is the most relevant conversion together with the LCA production. On the other hand, the epimer isoLCA and 7β-Hydroxy-5β-cholan-24-oic acid were the BAs preferred by gut microbes to produce MCBAs. The reason why the β-hydroxylated acid backbone is used to produce MCBAs while the β-dihydroxylated is not used is unknown. In general, the mono hydroxylated sterol backbone was the only structure used for the re-conjugations (Table 2). The production of β-hydroxy cholic acid esters were the most plausible reactions to explain the production of isomers and molecules with equal *m*/*z* and MS/MS fragments as well as the modifications observed in the retention times. According to the results, both the classical conversions and the new conjugation conversions change the chemical attributes by changing their hydrophobicity and therefore the BAs toxicity. BA hydrophobicity is determined by the number, position and orientation of the hydroxyl groups, as well as the amidation at the C-24 position. Regarding the magnitude of BA hydrophobicity, the order would be UDCA < CA < CDCA < DCA < LCA [37]. The results showed how the production of epimeric esters of LCA and 7β-Hydroxy-5β-cholan-24-oic acid clearly reduce the hydrophobicity of the original BAs and therefore their toxicity. This suggests that the impact of production of conjugates derived from LCA instead of CDCA may be a protective mechanism of bacteria against toxicity. The set of reactions that lead to reduce the number of β-hydroxy unconjugated BAs will produce a potentially more toxic pool of BAs.

This deep study of the presence of conjugates may be of great relevance. It has been described how theabrownin alters the intestinal microbes and suppresses bile-salt hydrolase (BSH) activity. This results in the increase in levels of the ileal conjugated bile acids and the production of the inhibition of the intestinal FXR-FGF15 signaling being potential anti-hypercholesterolemia and anti-hyperlipidemia therapies [38]. These reasons make relevant the re-evaluation of the new conjugate described in the present research in previous studies.

Therefore, the balance of epimerization, dehydroxylation and consequent re-conjugation will determine the global hydrophobicity of the BAs pool of the host and therefore the consequent reactions. The present in vitro study describes this new vision of BA conversion by the human gut microbiota in a reduced sample size, and shows the possibilities of how to explore the conversion diversity on populations affected by diseases related with changes in the pool of BAs.

To summarize, the importance of BAs is a fact, and they have been related with important pathologies such as non-alcoholic fatty liver diseases, irritable bowel syndrome, steatohepatitis, colorectal cancer and type-2 diabetes [39,40,41,42,43,44,45]. In addition, there is a very close health relationship with their functionality as signaling molecules interacting at specific cellular receptors [46]. Therefore, deep knowledge of how the gut microbiome affects the final pool of BAs of the host is essential in moving forward. This in vitro study suggests that we are facing a huge new BA conversion field and therefore the evaluation of the diversity of the MCBAs may help to determine the general hydrophobicity of the final set of BAs of the host and its consequent correct functional performance.

## 5. Conclusions

In conclusion, the importance of developing a greater understanding of the primary BAs and their interaction with the gut microbiota is key for unlocking the complete physiopathology of the BAs. The BAs influence has been reported to be related to relevant non-communicable diseases such as type-2 diabetes and colorectal cancer. Advancing the knowledge and understanding of these diseases is directly related with the relations of the binomial host microbes-BAs and the final pool produced. Therefore, the interaction between the gut microbes of the host and the primary BAs is a key factor in determining the final pool of BAs and their activity.

This study identified leucolithocholate ester and valolithocholate ester as new microbially conjugated bile acids. This find certified the capability of the gut microbes to produces esters of MCBAs on hydroxyls of the sterol backbone in addition to amides at the C24 acyl site and therefore highlighted the presence of a new family of MCBAs produced by esterification. These new MCBAs may modify the toxicity of the original unconjugated BA, alter the farnesoid X receptor signaling, and ultimately influence i the related diseases. The newly discovered microbially conjugated bile acids open a new study perspective in the BAs field and their influence on health.

## Figures and Tables

**Table 1:** BAs identified and confirmed of classical conversion trend of secondary BAs.

Compound Name	Formula	*m*/*z*	Rt	MS/MS Fragments	Collision E	Abundance abs *
Chenodeoxycholic acid (CDCA)	C_40_H_40_O_4_	391.2849	14.33	391.2864; 373.2752	40	1.9 × 10^5^
Isoursochenodexycholic acid (iUDCA)	C_40_H_40_O_4_	391.2849	11.66	391.2855; 373.2784	40	2.6 × 10^7^
3-oxo-chenodeoxycholic acid (3-oxoCDCA)	C_40_H_38_O_4_	389.2692	14.58	389.2708; 345.2819; 343.2666; 371.2643	40	2.1 × 10^5^
7-oxo-lithocholic acid (7-oxoLCA)	C_40_H_38_O_4_	389.2692	13.29	389.2722; 345.2814; 343.2668	40	3.1 × 10^4^
Lithocholic acid (LCA)	C_40_H_40_O_3_	375.2899	18.03	375.2944; 357.2838; 355.2679	50	9.5 × 10^7^
7α-Hydroxy-5β-cholan-24-oic acid	C_40_H_40_O_3_	375.2899	17.15	375.2947; 357.2947	50	1.4 × 10^7^
3-oxo-5β-cholan-24-oic-acid	C_40_H_38_O_3_	373.2743	18.4	373.2732; 355.2626	30	1.7 × 10^7^

* Absolute abundance observed after the incubation. Absolute abundance of commercial standard before incubation, correlated with 50 µM of standard, were 1.39 × 10^9^ and 1.45 × 10^8^ for CDCA and 3-oxoCDCA respectively.

**Table 2:** Microbially conjugated BAs identified and confirmed.

Compound Name	*m*/*z*	Formula	Rt	MS/MS Fragment
Leucolithocholic; Leucolithocholate ester	488.3754	C_30_H_51_NO_4_	18.50	488.3739; 130.0872
Leucolithocholic; Leucolithocholate ester	488.3735	C_30_H_51_NO_4_	18.17	488.3813; 444.3902; 130.0867
Isoleucolithocholic; Isoleucolithocholate ester	488.3745	C_30_H_51_NO_4_	17.20	488.3808; 444.3317; 130.0875
Valolithocholic; Valoisolithocholic; Valoisolithocholate ester	474.3602	C_29_H_49_NO_4_	17.15	474.3640; 430.3761; 116.0709
Valolithocholic; Valoisolithocholic; Valoisolithocholate ester	474.3591	C_29_H_49_NO_4_	16.80	474.3650; 430.3731; 116.0714
Valolithocholic; Valoisolithocholic; Valoisolithocholate ester	474.3548	C_29_H_49_NO_4_	16.16	474.3638; 430.3730; 116.0713
Valolithocholic; Valoisolithocholic; Valoisolithocholate ester	474.3589	C_29_H_49_NO_4_	15.48	474.3643; 430.3727; 116.0716
Valolithocholic; Valoisolithocholic; Valoisolithocholate ester	474.3584	C_29_H_49_NO_4_	14.83	474.3652; 430.3729; 116.0712
Triptophano-dioxochenodeoxycholic	573.3340	C_35_H_46_N_2_O_5_	15.60	N/D
Leucochenodeoxycholic; isolecochenodeoxycholic; Leucolithocholate ester	504.3694	C_30_H_51_NO_5_	13.90	N/D
Prololithocholic; Prololithocholate ester; valo-oxolithocholic	472.3412	C_29_H_47_NO_4_	18.20	N/D
Alanolithocholic; Alanolithocholate ester; Serocholic acid; Serocholate ester	446.3285	C_27_H_45_NO_4_	15.58	N/D
Alanolithocholic; Alanolithocholate ester; Serocholic acid; Serocholate ester	446.3269	C_27_H_45_NO_4_	15.90	N/D
Alanolithocholic; Alanolithocholate ester; Serocholic acid; Serocholate ester	446.3261	C_27_H_45_NO_4_	15.92	N/D
Arginolithocholic; Arginolithocholate ester	531.3759	C_30_H_52_N_4_O_4_	15.50	N/D
Lysocholic acid; Lysocholate ester	487.3930	C_30_H_52_N_2_O_3_	14.89	N/D
Lysocholic acid; Lysocholate ester	487.3933	C_30_H_52_N_2_O_3_	14.28	N/D
Threonocholic acid; Threonocholate ester	460.3432	C_28_H_47_NO_4_	15.15	N/D

Note: All MS/MS fragment were acquired at 20, 30 and 40 ev. N/D: No data.

## Data Availability

The authors confirm that the data supporting the findings of this study are available within the article. The raw data was deposited in Metabolights (https://www.ebi.ac.uk/metabolights/) repository under the accession numbers MTBLS4140 (accessed on 1 March 2022). Here you can find the link to access the study: https://www.ebi.ac.uk/metabolights/reviewer1a1b88ae-f41e-4ae9-a113-e42283b4b002.
